# Evolutionary and biogeographic history of the subfamily Neoplecostominae (Siluriformes: Loricariidae)

**DOI:** 10.1002/ece3.368

**Published:** 2012-08-29

**Authors:** Fábio F Roxo, Cláudio H Zawadzki, Markos A Alexandrou, Guilherme J Costa Silva, Marcio C Chiachio, Fausto Foresti, Claudio Oliveira

**Affiliations:** 1 Laboratório de Biologia e Genética de Peixes, Departamento de Morfologia, Universidade Estadual Paulista, UNESPBotucatu, SP, Brazil; 2 Nupélia, Universidade Estadual de Maringá, UEMMaringá, PR, Brazil; 3 Department of Ecology, Evolution and Marine Biology, University of California Santa BarbaraSanta Barbara, California; 4 Laboratório de Ictiologia, Departamento de Zoologia e Botânica, Universidade Estadual Paulista, UNESPSão José do Rio Preto, SP, Brazil

**Keywords:** Biogeography, catfish, freshwater, Loricariidae, molecular systematics, *Neoplecostomus*, Neotropics

## Abstract

Freshwater fish evolution has been shaped by changes in the earth's surface involving changes in the courses of rivers and fluctuations in sea level. The main objective of this study is to improve our knowledge of the evolution of loricariids, a numerous and adaptive group of freshwater catfish species, and the role of geological changes in their evolution. We use a number of different phylogenetic methods to test the relationships among 52 representative taxa within the Neoplecostominae using 4676 bps of mitochondrial and nuclear DNA. Our analysis revealed that the subfamily Neoplecostominae is monophyletic, including *Pseudotocinclus*, with three lineages recognized. The first lineage is composed of part of *Pareiorhina rudolphi*, *P*. cf. *rudolphi*, and *Pseudotocinclus*; the second is composed of *Isbrueckerichthys*, *Pareiorhaphis, Kronichthys*, and the species *Neoplecostomus ribeirensis*; and the third is composed of *Pareiorhina carrancas*, *P*. cf. *carrancas*, *Pareiorhina* sp. 1, a new genus, and all the species of the genus *Neoplecostomus*, except *N. ribeirensis*. The relaxed molecular clock calibration provides a temporal framework for the evolution of the group, which we use for a likelihood-based historical biogeographic analysis to test relevant hypotheses on the formation of southeast Brazil. We hypothesize that headwater capture events and marine regressions have shaped the patterns of distribution within the subfamily Neoplecostominae throughout the distinct basins of southeast Brazil.

## Introduction

The rivers of the Neotropics are hugely diverse, estimated to contain more than 7000 fish species (Eschmeyer 2011). Interestingly, most Neotropical ichthyofauna diversified within a comparatively short timeframe (post K/T boundary) (Albert and Reis 2011). The drainages of southeast Brazil, comprising the Upper Rio Paraná Basin and coastal rivers (Rio Paraiba do Sul and Rio Ribeira de Iguape), are among the most species-rich regions with regard to freshwater fish (Abell et al. 2008). Among Neotropical freshwater fishes lineages, the Loricariidae represent the largest family with 973 species recognized (Eschmeyer 2011) and at least 300 waiting to be described (Reis et al. 2003). Within Loricariidae, Neoplecostominae include 37 comparatively small-bodied species (Eschmeyer 2011) in the genera *Kronichthys*, *Isbrueckerichthys*, *Pareiorhaphis*, *Pareiorhina*, *Neoplecostomus*, and *Pseudotocinclus* (Montoya-Burgos et al. 1998; Armbruster 2004; Chiachio et al. 2008). Neoplecostominae representatives are restricted to the Southern and Southeastern regions of Brazil, making them an ideal group to test hypotheses of historical biogeography relevant to these regions. These fish are found in small-to-medium-sized streams with clear shallow water (commonly <1 m deep), moderate-to-strong currents, on loose stones, and sandy substrates (Langeani 1990; Pereira and Reis 2002). The Neoplecostominae have a long and complex history of taxonomy and systematics, with morphological and molecular studies focusing on evolution of the subfamily and also in Loricariidae as a whole (Eigenmann and Eigenmann 1890; Regan 1904; Gosline 1947; Isbrücker 1980; Howes 1983; Schaefer 1987; Montoya-Burgos et al. 1998; Armbruster 2004; Pereira 2005; Reis et al. 2006; Chiachio et al. 2008; Cramer et al. 2008, 2011). However, the historical evolutionary relationships among endemic species from the southeast of Brazil and the mechanisms by which they diversified in space and time remain understudied. This has been a challenge for generations of evolutionary biologists, mainly because of the difficulties in reconstructing the complex hydrogeological processes that have shaped the Neotropics (Ribeiro et al. 2006; Albert and Reis 2011).

Despite of the complexity of biotic spatiotemporal distribution, in the last few decades, our understanding of the historical biogeography of Neotropical freshwater fishes has improved with the advent of plethora of phylogenetic, paleontological, paleoclimatic, and paleoenvironmental data (Lundberg and Chernoff 1992; Casciotta and Arratia 1993; Gayet 2001; Gayet and Meunier 2003; Lundberg and Aguilera 2003; Lundberg 2005; Sanchez-Villagra and Aguilera 2006; Malabarba and Lundberg 2007; Malabarba and Malabarba 2008; Albert and Reis 2011). Abell et al. (2008), in an extensive synthesis on freshwater fish endemism throughout the world, proposed the following four eco-regions for southeast Brazil: the coastal drainages of Paraiba do Sul, Ribeira de Iguape and Southeastern Mata Atlântica, and the interior drainage of São Paulo State, known as Upper Paraná. Such eco-regions are supported by published phylogenetic data indicating distinct ichthyofaunal divisions between the littoral drainages and Upper Paraná (Montoya-Burgos 2003; Hubert and Renno 2006). In a recent study, Chiachio et al. (2008) tested biogeographic hypotheses involving the subfamilies Neoplecostominae, Otothyrinae, and Hypoptopomatinae using two dispersal-extinction-cladogenesis models to infer the spatiotemporal distribution of these fishes. Maximum-likelihood (ML) reconstructions of ancestral ranges indicated a marked division between the Amazonian origin of the Hypoptopomatinae and the eastern coastal Brazil + Upper Paraná origin of the Neoplecostominae and Otothyrinae.

**Figure 1:**
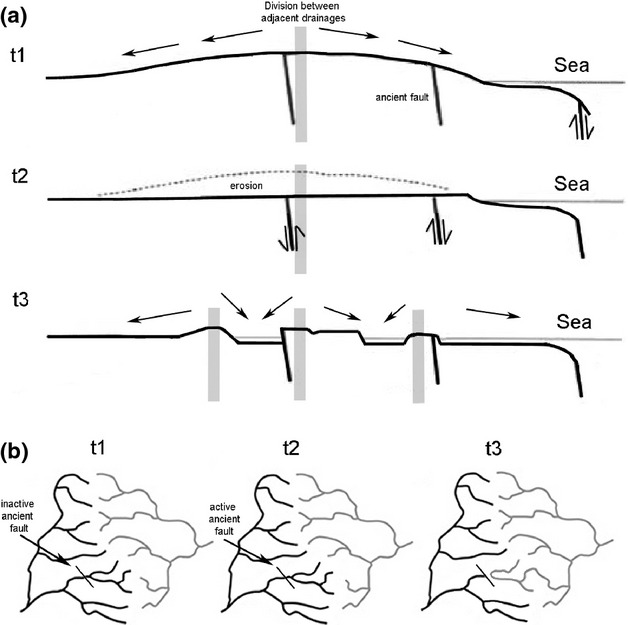
(a) Scheme activation of ancient faults and the erosive process in Serra do Mar formation (arrow indicate the direction of the flow); (b) Scheme of the headwater captures resulted of the activation ancient faults and erosive process. Modified from Almeida and Carneiro (1998) and Albert and Reis (2011).

Ribeiro (2006) has provided an overview of the principal aspects of geological history along the continental margin of eastern South America and explained the biogeographic history of fish fauna from the Brazilian crystalline shield, focusing mainly on the coastal drainages of eastern Brazil. According to Ribeiro (2006), many cladogenetic events associated with tectonics and erosive processes (which are still active today across eastern South America) may be influencing ichthyofaunal distribution and speciation (Fig. 1). Ribeiro (2006) described river headwater capture events as a complex process associated with the main fault systems, which are more susceptible to erosion. In this process, portions of the tributaries of a river in one hydrographic basin are “captured” by adjacent basins resulting in isolated populations (Fig. 1). This process occurred several times in the formation of Southeastern Brazilian shield. Albert and Reis (2011) suggested that between 15 and 28 million years ago several head water captures occurred in the Upper Paraná basin and coastal rivers in Tremembe formation, whereas Ribeiro (2006) suggested the same for the Ponta Grossa Arch formation. Also, Montoya-Burgos (2003) studying the biogeography of the loricariid genus *Hypostomus* suggested that headwater captures occurred between the Upper Rio Paraná and Eastern Coastal Rivers 4.2 million years ago. These vicariant processes were followed by allopatric divergence and speciation within *Hypostomus*. A similar hypothesis has been proposed by Weitzman et al. (1988) working with Glandulocaudini species.

Several authors have suggested that species diversity in Brazil could also be related to the occurrence of sea level fluctuations that have allowed the dispersal between neighboring rivers via delta connections or coastal marshes (Hoorn 1993; Monsch 1998; Hernández et al. 2005; Roddaz et al. 2005; Rebata et al. 2006; Hoorn and Wesselingh 2010). Montoya-Burgos (2003) used this class of events to explain the split between the lineages inhabiting the eastern coastal rivers and those inhabiting Amazon drainages for species of the genus *Hypostomus* between 5 and 6 million years ago. Bloom and Lovejoy (2011) further suggested that vast areas of South America, particularly, the Amazon basin, have relatively low elevations (<100 m) and are thus likely to be impacted by sea level fluctuations. Moreover, from the middle of the Miocene to the present, the climate has become colder and has undergone periodic climatic oscillations that were responsible for marine transgression and regression. Albert and Reis (2011) suggest that large marine regressions occurred between 76–80, 66–71, 45–50, 35–42, 23–34, 9–12, and from 3.5 million years ago to the present when climatic oscillations intensified. The main factors responsible for climatic oscillation are atmospheric CO_2_ concentration, thermohaline circulation, the positions of the continental landmasses, and the tilt of the planet's axis (Budyko 1969; Petit et al. 1999; Rahmstorf 2003; Rothman et al. 2003).

In this study, we use a phylogenetic approach to reconstruct a species tree for the Neoplecostominae and infer their age, origin, and ancestral dispersal routes throughout southeastern Brazil. We test relevant biogeographic hypotheses (Table 1) using a dispersal-extinction-cladogenesis approach, and discuss patterns likely to have influenced the spatiotemporal distribution of Neotropical fish fauna in the Southeastern part of Brazil.

**Table 1:** Events which have influenced diversification of fish species in Southeastern Brazil

Event	Predictions	Methods used	References
1. Boundary displacements between the Upper Paraná and the Littoral Drainages	Sister lineages from different endemic drainages will be monophyletic	Phylogeny	Present study
2. Head water capture events between major drainages	(i) Clades (intraspecific lineages or species groups) will not be monophyletic for a given drainage;	Phylogeny and molecular clock	Weitzman et al. (1988), Ribeiro et al. (2006), Lundberg (1998), Montoya-Burgos (2003), Hubert and Renno (2006)
	(ii) lineages in a given watershed will be nested within the phylogeny of a larger clade located in a different watershed with contiguous headwater.		
3. Miocene, Pliocene and Pleistocene climate oscillation	(i) Delta connection in coastal rivers in marines regression possibility the dispersal of species to others drainages.	Phylogeny and Molecular clock	Montoya-Burgos (2003), Hoorn and Wesselingh (2010), Abell et al. (2008), Hubert and Renno (2006), Ab'Saber 1979), Colinvaux et al. (2000), Petit et al. 1999), Wilkinson et al. (2006)

## Material and Methods

### Sampling

**Figure 2:**
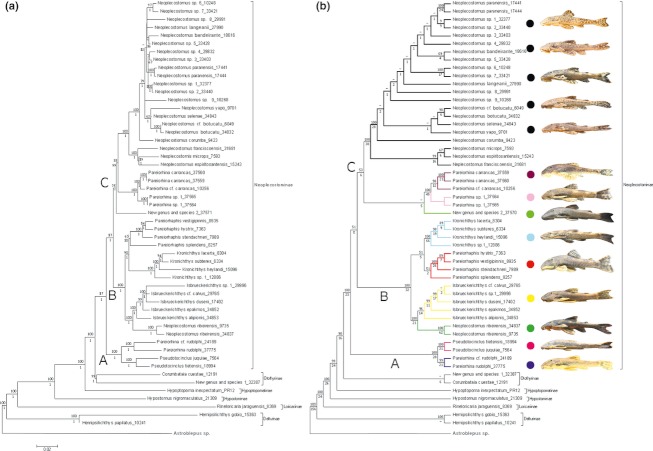
(a) Majority-rule consensus tree obtained in ML and BI analyses. Numbers above branches are bootstrap values from 1000 bootstrap pseudoreplicates obtained from ML analysis. Bootstrap values below 50% (−) are not shown. Numbers below branches are posterior probabilities obtained in the BI analysis. (b) Single most parsimonious tree obtained in the MP analysis (CI = 0.509, RI = 0.682). Numbers above branches are bootstrap values from 1000 bootstrap pseudoreplicates. Numbers below branches are Bremer decay support values. Bootstrap values below 50% (-) are not shown.

All fishes collected for this study (Table S1) were collected in accordance with Brazilian laws, under a permanent scientific collection license in the name of Dr. Claudio Oliveira. Additionally, our laboratory has special federal permission to keep animals and tissues from a public collection under our care. To work with the animals, we follow all the ethical prescriptions stated by our internal committee of ethic involving animal experiments (protocol number 388). The species were collected using hand nets, from a variety of locations across southeastern Brazil (Fig. 2, Table S1). After collection, the animals, to do not suffer any kind of pain, were anesthetized using 1% benzocaine in water and either preserved in 95% ethanol for molecular studies or fixed in 10% formaldehyde for morphological studies. Vouchers of all sequenced samples were deposited in the collection of either the Laboratório de Biologia e Genética de Peixes (LBP), Departamento de Morfologia, Instituto de Biociências, Universidade Estadual Paulista, Botucatu, São Paulo, Brazil, the Núcleo de Pesquisas em Limnologia, Ictiologia e Aquicultura (NUP), Universidade Estadual de Maringá, Paraná, Brazil, or the Museum of Natural History of the City of Geneva (MHNG), Geneva, Switzerland. In the course of this study, several new species and two new genera were found and will be further described in subsequent publications.

### DNA sequencing

Total DNA was extracted from ethanol-preserved muscle, fin, and liver samples using the Wizard Genomic DNA Purification Kit (Promega, Madison, Wisconsin). Partial sequences of 16S rRNA (700 bp), 12S rRNA (900 bp), cytochrome c oxidase subunit I (COI, 700 bp), cytochrome b (Cytb, 900 bp), and F-reticulon 4 (1900 bp) were amplified using polymerase chain reaction (PCR) with the primers described in Table S2. The primer concentration was 5 pmol/μL. Mitochondrial genes were amplified with a total volume of 25 μL for 35 cycles (30 sec at 95°C, 45 sec at 48–54°C, and 80 sec at 72°C). The nuclear markers were amplified in two PCR experiments; the first amplification using the primers Freticul4-D and Freticul4-R with a total volume of 12.5 μL for 37–40 cycles (30 sec at 95°C, 30 sec at 48°C, and 135 sec at 72°C); and the second amplification using the primers Freticul4 D2, Freticul4 R2, and Freticul4 iR with a total volume of 12.5 μL for 37–40 cycles (30 sec at 95°C, 30 sec at 53–54°C, and 135 sec at 72°C). The PCR was amplified using the Gotaq commercial kit (Promega). After that, the products were identified on a 1% agarose gel. The PCR products were purified using ExoSap-IT® (USB, Affymetrix Corporation, Cleveland, Ohio) following the manufacturer's instructions. The purified PCR products were used to make a sequencing PCR using the BigDye™ Terminator v 3.1 Cycle Sequencing Ready Reaction Kit (Applied Biosystems- Life Technologies do Brasil Ltda, Vila Guarani, SP, Brazil). Subsequently, the amplified DNA was purified again and loaded on a 3130-Genetic Analyzer automatic sequencer (Applied Biosystems). All sequences obtained in this study were deposited in the GenBank (Table S1).

#### Phylogenetic analyses

An *Astroblepus* sp. (Astroblepidae) was used to root the Neoplecostominae phylogeny. Additionally, samples of Delturinae (*Hemipsilichthys gobio* and *H. papillatus*), Loricariinae (*Rineloricaria jaraguensis*), Hypoptopomatinae (*Hypoptopoma inexpectatum*), Otothyrinae (*Corumbataia cuestae*), and Hypostominae (*Hypostomus nigromaculatus*) were included in the analysis as additional outgroups. Sequences from databases was not included in our analysis because only sequences of the gene F-reticulon 4 are available, and the increase in gaps number should reduce the phylogenies accuracy like suggested by Dwivedi and Gadagkar (2009). Also, we have a great sample of Neoplecostominae species and that was considered monophyletic by Chiachio et al. (2008) using one of the molecular markers included in our analysis, F-reticulon 4. All individual sequences for each species were initially analyzed using the software BioEdit 5.0.9 (Hall 1999) and a consensus sequences were obtained. Afterward, all sequences were independently aligned using the software Muscle (Edgar 2004). Nucleotide variation, substitution patterns, and genetic distances were examined using MEGA 5.0 (Tamura et al. 2007). All different nucleotide substitution models were tested using Modeltest 3.7 (Posada and Crandall 1998) to 19 different partitions of the matrix. For each phylogenetic program analysis, a related model was used when the program do not present the nucleotide substitution models found for Modeltest. The partition and the models utilized for each one are shown in Table S3.

Maximum-likelihood analyses were performed using RAxML Web-Servers (Stamatakis et al. 2008). It implements a faster algorithmic of heuristic search with bootstrap pseudoreplicates (RBS). Bootstrap resampling (Felsenstein 1985) was applied to assess support for individual nodes using 1000 replicates. Random starting trees were used for each independent ML tree search and all other parameters were set on default values. All ML analyses were conducted under GTR, as RAxML only applies this model, using or not G according to Modeltest 3.7 (Posada and Crandall 1998) search (Table S3).

Bayesian inference (BI) (Huelsenbeck and Ronquist 2001) was performed evaluating alternative tree topologies through the estimation of posterior probabilities using MrBayes v.3.0 (Ronquist and Huelsenbeck 2003). MrBayes implements three main types of nucleotide substitution models and they are selected by setting the number of substitution types using lset nst to 1, 2, or 6. This parameter was selected to each matrix partition (Table S3). Four chains were run simultaneously for 50,000,000 generations and every 1000th generation, a tree was sampled. The above analysis was performed twice. The distribution of log likelihood scores was examined to determine stationarity for each search and to decide if extra runs were required to achieve convergence, using the program Tracer 1.4 (Rambaut and Drummond 2007a). All sampled topologies beneath the asymptote (5,000,000 generations) were discarded as part of a burn-in procedure, and the remaining trees were used to construct a 50% majority-rule consensus tree in Paup* (Swofford 2003).

Maximum-parsimony (MP) was performed using the software TNT (Goloboff et al. 2008). No a priori weighting or ordering of character states was used and gaps were treated as missing data. Phylogenies were constructed under the “new technology search” methodology (Goloboff 1996, 1999), using the options “sectorial search”, “ratched”, “drift”, and “tree fusing” with their default values and employing a driven search with initial level setting at level 100 and checking level at every two hits. Consistency and retention indexes (RIs) were calculated with the script “stats” of TNT. Clade robustness was assessed using 1000 bootstrap (B) pseudoreplicates (Felsenstein 1985) with the same parameters cited above. Bremer support values (BS) (Bremer 1988) were calculated with the script “bremer” script for TNT.

### Molecular clock and historical biogeography

The uncorrelated relaxed molecular clock (lognormal) was calibrated using BEAST (Bayesian evolutionary analysis sampling trees) v1.6.2, (Drummond and Rambaut 2007) on the same dataset used above with the same partitioning scheme (Table S3). The models utilized for each partition are shown in Table S3. We used the origin of Loricariidae family (85–90 million years ago), as estimated by Lundberg et al. (2007), for our calibration point at the root of the tree. This was implemented with a lognormal prior offset of 85 million years ago with a mean and standard deviation of 1. We used a Birth–Death model for speciation likelihood and a starting tree obtained from ML analysis. The analysis was run for 100 million generations and sampled every 1000th generation. Stationarity and sufficient mixing of parameters (ESS > 200) was checked using Tracer v1.5 (Rambaut and Drummond 2007a). A consensus tree was built using TreeAnnotator v1.6.2 (Rambaut and Drummond 2007b).

Maximum-likelihood inference of geographic range evolution was performed using the Dispersal-Extinction-Cladogenesis (DEC) as implemented in Lagrange v 2.0 (Ree et al. 2005; Ree and Smith 2008). The DEC model specifies instantaneous transition rates between discrete distribution ranges along the branches of a phylogenetic tree and uses it to assess likelihoods of ancestral distribution ranges at cladogenesis events (Ree et al. 2005; Ree and Smith 2008). We used the unconstrained DEC model because the geographic region of interest was only divided into two areas (interior and littoral drainages). We classified the divisions of endemic areas according to the eco-region classifications of Abell et al. (2008), Montoya-Burgos (2003), and Hubert and Renno (2006). “Littoral drainages” was a classification composed of the following eco-regions: Northeastern Mata Atlântica, Paraiba do Sul, Ribeira do Iguape, Southeastern Mata Atlântica, and Fluminense. “Interior drainages” was composed of Upper Paraná, Iguassu, and São Francisco.

## Results

### Phylogeny of the Neoplecostominae

Partial sequences of four mitochondrial genes (12S rRNA, 16SrRNA, COI, Cytb) and one nuclear gene (F-reticulon 4) were obtained from 52 specimens representing 47 loricariid species and one Astroblepidae species (Table S1). The combined sequence data resulted in a matrix with 4676 base pairs (bp), out of which 2676 were conserved and 1155 were parsimony-informative. This matrix was used to perform all phylogenetic and biogeographic analyses and was partitioned by gene and coding positions into 19 sections (Table S3). These data were not saturated as observed by transitions/transversions rate (3.2) compared with genetic distance.

A Bayesian analysis was performed with 50,000,000 generations that resulted in 50,001 trees, of which the first 5000 were discarded and the remaining 45,001 were used to construct the consensus tree (Fig. 2a). The MP analysis with the “new technology search” (Goloboff 1996, 1999) resulted in a single most parsimonious tree (Fig. 2b) with a consistency index = 0.509 and a RI = 0.682. All nodes were supported by Bremer indexes with values ranging from 1 to 156, and most of the nodes were also supported by high bootstrap values.

Figure 2(a) (ML and BI analyses) and Figure 2(b) (MP) illustrate that Neoplecostominae is only monophyletic when including the genus *Pseudotocinclus*, with considerable statistical support (BS = 100 with ML and P = 1 with BI, BS = 100 and B = 23 with MP).

Three lineages are recognized within Neoplecostominae. The first lineage (clade A) is composed of *Pareiorhina rudolphi, P*. cf. *rudolphi*, and *Pseudotocinclus* (BS = 100 with ML and P = 1 with BI, BS = 100 and B = 28 with MP), and is distributed across both eco-regions, littoral, and interior. The second lineage (clade B) is composed of *Isbrueckerichthys*, *Pareiorhaphis, Kronichthys*, and the species *Neoplecostomus ribeirensis* (BS = 100 with ML and P = 1 with BI, BS = 100 and B = 32 with MP) and is almost exclusive to littoral. The third lineage (clade C) is composed of *Pareiorhina carrancas*, *P*. cf. *carrancas*, *Pareiorhina* sp. 1 a new genus, and all species of the genus *Neoplecostomus*, except *Neoplecostomus ribeirensis* (BS = 75 with ML and P = 1 with BI, BS = 51, and B = 5 with MP).

### Relaxed molecular clock and historical biogeography

**Figure 3:**
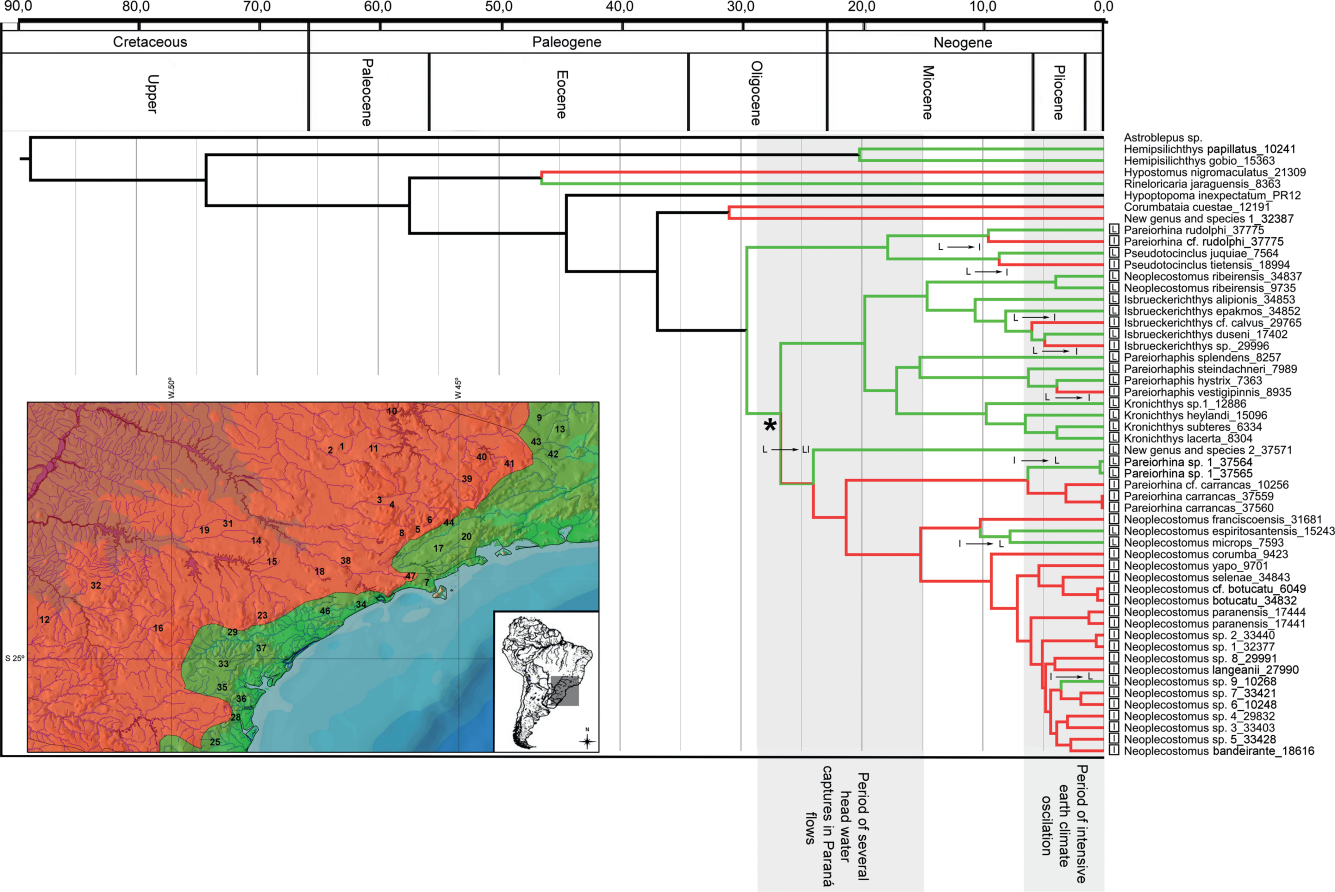
The red coloration in the figure indicate the interior drainages and green the littoral drainages in southeastern of Brazil. Numbers in the map refer collection points listed in the Supplemental Material 1. The tree is the beast chronogram tree from 100 million generations, showing diverge age in subfamily Neoplecostominae, calibrated on the origin of Loricariidae (85–90 million years ago) based on Lundberg et al. (2007). Scale = millions of years before present. The Maximum-likelihood inference of geographic range evolution in Neoplecostominae, performed using Dispersal-Extinction-Cladogenesis implemented in Lagrange v 2.0. L, littoral; I, interior is indicated in the tree. Asterisk indicates when the ancestral of clade C reaches the interior between 26.7 (15.4–38.1) million years ago.

The mean substitution rate for the Neoplecostominae dataset estimated using Beast was 0.808% per MY. The origin of Neoplecostominae was 36.9 million years ago (95% HPD: 22.3–52.1) with a crown age estimate for the *Neoplecostomus* of 21.3 million years ago (95% HPD: 11.6–31.1) (Fig. 3). Within Neoplecostominae, clade A originated 29.5 million years ago (95% HPD: 17.4–42.1). The ancestral lineage of *Pseudotocinclus tietensis* reach the interior from littoral drainages 8.6 million years ago (95% HPD: 2.7–15.4), and *Pareiorhina* cf. *rudolphi* 1 9.5 million years ago (95% HPD: 3.0–17.0). Results of Lagrange and molecular clock analysis suggest that the splitting between clade B and C is 26.7 million years ago (95% HPD: 15.4–38.1, Fig. 3), and during that time the ancestral of clade C reached the interior from littoral drainages (Fig. 3). Within clade B more recent lineages also reached the interior from littoral drainages: that is the ancestor of *Pareiorhaphis vestigipinnis* 3.8 million years ago (95% HPD: 1.0–7.3), the ancestor of *Isbrueckerichthys* sp. 1 4.8 million years ago (95% HPD: 2.3–7.8), the ancestor of *I*. cf. *calvus* 5.9 million years ago (95% HPD: 2.9–9.5), and the ancestor of *I. alipionis* 10.6 million years ago (95% HPD: 5.5–16.5). Also, lineages from interior reached the littoral drainages between 3.5 and 24.0 million years ago: ancestral node of New genus and species 2 24.0 million years ago (95% HPD: 13.3–34.5), *Pareiorhina* sp. 1 6.2 million years ago (95% HPD: 2.3–11.2), ancestral node of *Neoplecostomus* sp. 9 3.5 million years ago (95% HPD: 1.6–5.5), the ancestor of *Neoplecostomus microps*, and *N. espiritosantensis* 10.2 million years ago (95% HPD: 4.7–16.5). Thus, considering our Lagrange results (Fig. 3), we observed more lineages of Neoplecostominae reaching the interior from littoral drainages (six events) than littoral from interior drainages (four events).

## Discussion

### Headwater captures dispersal hypothesis

Our analysis suggests that the ancestor of the Neoplecostominae originated in littoral drainages 36.9 (22.3–52.1) million years ago (Fig. 3), based on the results from our relaxed uncorrelated molecular clock analysis. The same coastal origin was proposed by Chiachio et al. (2008). Ribeiro (2006) proposed some interesting biogeographic patterns exhibited by the freshwater ichthyofauna on the Brazilian crystalline shield and the Atlantic coastal drainages. The first pattern was defined by sister-group relationships between the endemic ichthyofauna of the Brazilian coastal drainages and adjacent shield that includes intermediate degree of inclusive and both sister-clades underwent some radiation. Ribeiro (2006) postulated that the members of genera *Lignobrycon*, *Rhinelepis, Spintherobolus*, and *Triportheus*, the tribes Aspidoradini and Glandulocaudini, and the subfamilies Cheirodontinae and Sarcoglanidinae are examples of this pattern. This distribution of species is similar to that found in our results to subfamily Neoplecostominae, in which species of clade B are almost exclusive to littoral drainages and those of clade C are almost exclusive to the interior.

Another pattern of distribution of fish fauna recognized by Ribeiro (2006) is related to the families Doradidae and Trichomycteridae, in which a taxon of ancient fish inhabiting littoral rivers is a sister group to a much more inclusive, derived, and widespread taxon. Additionally, all species of the subfamily Delturinae (which has been suggested by Armbruster 2004 as sister group of all other loricariids, excluding the genus *Lithogenes*) are distributed in littoral rivers (Rio Paraíba do Sul and Rio Doce). This reinforces the hypothesis that the coastal drainages of Southeastern Brazil represent an important origin for many lineages of Neotropical catfishes. However, this cannot be considered a general rule applicable to the origin of all Neotropical fishes. Weitzman et al. (1988), while working with morphological phylogeny for Glandulocaudini, proposed an opposite evolutionary scenario where ancestral species inhabit the Paraguay and Parana basins (interior), whereas more derived species are distributed along coastal rivers (littoral). However, Weitzman's hypothesis for the Glandulocaudini has yet to be tested using additional data and fossil-calibrated analysis.

Our results also suggested that many species of Neoplecostominae are distributed throughout elevated regions of Southeastern Brazil (Fig. 3). Species of this subfamily are known to require waters with high oxygen concentrations (Langeani 1990), and it is possible that they have difficulty adapting to rivers in lower elevations where the oxygen concentration is low. Hoorn (1993) discusses two great marine incursions that occurred between 17 and 20 million years ago and another between 10 and 12 million years ago. These incursions most likely inundated lower elevation rivers of southeastern Brazil up to the La Plata basin (Hoorn 1993). This suggests that the ancestral Neoplecostominae species inhabiting rivers that were inundated by those marine incursions are likely to have gone extinct; if not entirely, then perhaps at the local population level. Such events may have also prevented subsequent lineages from successfully recolonizing and invading rivers affected by past marine incursions.

Within the Neoplecostominae, our phylogenetic analyses identified three lineages. The first is clade A, with species distributed throughout both littoral and interior drainages, originating 29.5 (17.4–42.1) million years ago. The second is clade B, with species almost exclusive to littoral drainages, and the third is clade C, which is almost exclusively distributed in interior drainages. Clades B and C form sister groups and originated 26.7 (15.4–38.1) million years ago. Albert and Reis (2011) reported that several headwater capture events occurred between 15 and 28 million years ago among the Rio Tietê, Rio Paraíba do Sul, Rio São Francisco, and Rio Ribeira de Iguape basins. This process is likely to have influenced ancestral fish movements throughout adjacent drainages, such as the clade C, reached the interior from littoral drainages (Fig. 3), and ancestral lineages reached the littoral from interior drainages (e.g., the ancestor of a new genus and species 2 24.0 (13.3–34.5) million years ago). Our results also suggest that more recent lineages of Neoplecostominae have reached the littoral from interior through head water captures, such as the ancestor of *Pseudotocinclus tietensis* 8.6 (2.7–15.4) million years ago and that of *Pareiorhina* cf. *rudolphi* 9.5 (3.0–17.0) million years ago. Ribeiro (2006) related that the activations of the ancient faults, responsible to head waters captures, occurred several times in the Serra do Mar formation, an area of southeast Brazil where these species are distributed. Riccomini and Assumpção (1999) suggested that the Continental Rift of Southeastern Brazil underwent significant restructuring (upper Miocene, Pliocene, Pleistocene, and Holocene), resulting in the reactivation of ancient structural lines. This finding reinforces the hypothesis that southeast Brazil has undergone intensive geological activity and that the activations of ancient faults could have resulted in headwater captures between adjacent drainages during several periods of its geological history.

Within clade B, we analyzed five species of the genus *Isbrueckerichthys*, and the results from the Lagrange analysis (Fig. 3) suggest that the ancestor of this genus inhabited littoral drainages. However, *Isbrueckerichthys* sp. 1, *I*. cf. *calvus*, and *I. alipionis* inhabit interior drainages. This result suggests that headwater captures could be responsible for the current distribution of the species inhabiting interior drainages, with ancestral lineages reaching the interior from the littoral 4.8 (2.3–7.8), 5.9 (2.9–9.5), and 10.6 (5.5–16.5) million years ago. Montoya-Burgos (2003) previously suggested that headwater capture events occurring between coastal drainages and the Upper Rio Paraná (over 4.2 million years ago) have also influenced the current distribution of the genus *Hypostomus*.

For the genus *Pareiorhaphis*, we included only four of the 19 nominal species (Pereira et al. 2007, 2010) in the present study. However, the species not included herein occur throughout almost all portions of the Oriental Coastal Basin and only a few species are found in interior drainages. Specifically, our results suggested that *P. vestigipinnis* could have reached interior drainages 3.8 (1.0–7.3) million years ago, further supporting the hypothesis that headwater captures occurred several times in the geological evolution of southeastern Brazil. Species of the genus *Kronichthys* are all from littoral drainages, suggesting that this genus could have originated there.

Within clade C, our results suggest that *Neoplecostomus* originated in the middle Miocene 21.3 (11.6–31.1) million years ago (all species of *Neoplecostomus*, except *N. ribeirensis*) and is older than the origin of any other Neoplecostominae genus (see Fig. 3). Despite the genetic diversity found in *Neoplecostomus*, these species are known to be morphologically conserved and difficult to distinguish (Langeani 1990; Zawadzki et al. 2008). However, it remains unknown whether this morphological similarity is due to historical mixture between interior and littoral drainages that mixed populations. Our results suggest that after the colonization of interior drainages, some *Neoplecostomus* ancestors reached the littoral from interior drainages (most likely via headwater captures), such as the ancestor of *Neoplecostomus* sp. 9 around 3.5 (1.6–5.5) million years ago and the ancestor of *Neoplecostomus microps* and *N. espiritosantensis* around 10.2 (4.7–16.5) million years ago. Furthermore, two lineages closely related to the genus *Neoplecostomus* reached the littoral from interior drainages: the ancestral of the new genus and species 2 24.0 (13.3–34.5) million years ago and the ancestor of the *Pareiorhina* sp. 1 6.2 (2.3–11.2) million years ago.

### Earth climate oscillation dispersal hypothesis

The sea level and tectonic deformations of the continental platform have changed drastically in the last 100 million years ago, but more intensively in the last 10 million years ago as a result of climatic oscillations (Rossetti 2001; Miller et al. 2005; Müller et al. 2008; Santos et al. 2008; Zachos et al. 2008; Albert and Reis 2011). After the Middle Miocene Optimum Climate, the climate of the Earth changed quickly to a drier and colder resulting in the Antarctic Reglaciation (Petit et al. 1999; Zachos et al. 2008). It was a period of great climate oscillation that was intensified throughout the Pliocene, Pleistocene, and Holocene (Suguio et al. 1985) resulting in multiple regressions and transgression of marine waters (Hoorn 1993; Lovejoy et al. 1998; Lundberg 1998). In ages of marine regression, animals that lived in the coastal rivers might have had contact with adjacent drainages through river deltas. These connections would have been impossible during periods of high sea level (Montoya-Burgos 2003; Hoorn and Wesselingh 2010). This hypothesis can help to explain the great diversity of species distributed throughout the coastal drainages of Southeastern of Brazil and specifically the dispersal of Neoplecostominae genera through unconnected adjacent coastal drainages. Our results suggest that most of the lineages in Neoplecostominae appeared in the last 10 million years ago (Fig. 3), during a period of high variation in the sea level. These variations are often related to oscillation of climate in Miocene, Pliocene and Pleistocene periods (Ab'Saber 1979; Colinvaux et al. 2000; Wilkinson et al. 2006; Zachos et al. 2008). Such events also affect species living in the interior drainages and could have influenced the dispersal of Neoplecostominae species within the hydrographical basin. Montoya-Burgos (2003) also related this hypothesis of delta connections during marine regressions to explain dispersal of lineages of genus *Hypostomus* through adjacent coastal drainages in Amazon and northeastern of Brazil.

In conclusion, we hope that the previously discussed hypotheses can help in the conservation policies for freshwater fishes in southeastern Brazil, an area of vital importance in the evolution of Loricariidae and other Neotropical fish groups. Phylogenetic and biogeographical knowledge continues to be essential for the elucidation of the magnitude of species richness and species evolution and distribution patterns. However, as the mechanisms that drive the great diversification of Neotropical fish remain understudied and significant phylogenetic information is lacking for many groups occurring in eastern Brazil, this region still deserves significant research effort.

## Biosketches

Fábio F. Roxo works with molecular systematic and has a long-term interest in the analysis of Neotropical Fishes, with emphasis in the family Loricariidae.

Cláudio H. Zawadzki works with taxonomy and has a long-term interest classification of species of family Loricariidae.

## Supporting Information

**Table S1.** Species included in the present study. LBP = Laborató rio de Biologia e Genética de Peixes, Universidade Estadual Paulista. NUP = Núcleo de Pesquisas em Limnologia, Ictiologia e Aqüicultura, Universidade Estadual de Maringá. MHNG = Museum of Natural History of the City of Geneva. Asterisk indicates the geographic position of the city, which the samples were collected and not the collection site.

**Table S2.** Primers used in the present study to amplify partial sequences of 12S and 16S rRNA, cytochrome oxidase subunit I (COI), cytochrome B (CytB), and F-reticulon 4.

**Table S3.** Nucleotide substitution models for each partition used in the phylogenetic analyses of each program.

## References

[b1] Abell R, Thieme ML, Revenga C, Bryer M, Kottelat M, Bogutskaya N (2008). Freshwater ecoregions of the world: a new map of biogeographic units for freshwater biodiversity conservetion. Bioscience.

[b2] Ab'Saber AN (1979). Os mecanismos de desintegração das paisagens tropicais no Pleistoceno: efeitos paleoclimáticos do período Würm-Wisconsin no Brasil.

[b3] Albert JS, Reis RE (2011). Historical biogeography of Neotropical freshwater fishes.

[b4] Almeida FFM, Carneiro CDR (1998). Origem e evolução da Serra do Mar. Revista Brasileira de Geociências.

[b5] Armbruster JW (2004). Phylogenetic relationships of the sucker-mouth armored catfishes (Loricariidae) with particular emphasis on the Hypostominae and the Ancistrinae. Zool. J. Linn. Soc.

[b6] Bloom DD, Lovejoy NR, Albert JS, Reis RE (2011). The biogeography of marine incursions in South America. Historical biogeography of Neotropical freshwater fishes.

[b7] Bremer K (1988). The limits of amino acid sequence data in angiosperm phylogenetic reconstruction. Evolution.

[b8] Budyko MI (1969). Effect of solar radiation variation on climate of earth. Tellus.

[b9] Casciotta JR, Arratia G (1993). Tertiary cichlid fishes from Argentina and reassessment of the phylogeny of new world cichlids (Perciformes: Labroidei). Kaupia.

[b10] Chiachio MC, Oliveira C, Montoya-Burgos JI (2008). Molecular systematic and historical biogeography of the armored Neotropical catfishes Hypoptopomatinae and Neoplecostominae (Siluriformes: Loricariidae). Mol. Phylogenet. Evol.

[b11] Colinvaux PA, Oliveira De, Bush M (2000). Amazonian and Neotropical plant communities on glacial time-scales: the failure of the aridity and refuge hypothesis. Quatern. Sci. Rev.

[b12] Cramer CA, Liedke AMR, Bonatto LS, Reis RE (2008). The phylogenetic relationship of the Hypoptopomatinae and Neoplecostominae (Siluriformes: Loricariidae) as inferred from mitochondrial cytochrome c oxidase I sequences. Bull. Fish Biol.

[b13] Cramer CA, Bonatto SL, Reis R (2011). Molecular Phylogeny of the Neoplecostominae and Hypoptopomatinae (Siluriformes: Loricariidae) using multiple genes. Mol. Phylogenet. Evol.

[b14] Drummond A, Rambaut A (2007). BEAST: bayesian evolutionary analysis by sampling trees. BMC Evol. Biol.

[b15] Dwivedi B, Gadagkar SR (2009). Phylogenetic inference under varying proportions of indel-induced alignment gaps. BMC Evol. Biol.

[b16] Edgar RC (2004). Muscle: a multiple sequence alignment method with reduced time and space complexity. BMC Bioinformatics.

[b17] Eigenmann CH, Eigenmann RS (1890).

[b18] Eschmeyer W (2011). Catalog of fishes. http://www.calacademy.org/research/ichthyology/catalog.

[b19] Felsenstein J (1985). Confidence limits on phylogenies: an approach using the bootstrap. Evolution.

[b20] Gayet M (2001). A review of some problems associated with the occurrences of fossil vertebrates in South America. J. S. Am. Earth Sci.

[b21] Gayet M, Meunier FJ, Arratia G, Kapoor BG, Chardon M, Diogo R (2003). Palaeontology and palaeobiogeography of catfishes. Catfishes.

[b22] Goloboff PA (1996). Methods for faster parsimony analysis. Cladistics.

[b23] Goloboff PA (1999). Analyzing large data sets in reasonable times: solutions for composite optima. Cladistics.

[b24] Goloboff PA, Farris JS, Nixon KC (2008). TNT, a free program for phylogenetic analysis. Cladistics.

[b25] Gosline WA (1947). Contributions to the classification of the loricariid catfishes. Arquivos do Museu Nacional do Rio de Janeiro.

[b26] Hall TA (1999). BioEdit: a user-friendly biological sequence alignment editor and analysis program for Windows 95/98/NT. Nucleic Acids Symp. Ser.

[b27] Hernández RM, Jordan TE, Farjat AD, Echavarria L, Idle-man BD, Reynolds JH (2005). Age, distribution, tectonics, and eustatic controls of the Paranense and Caribbean marine transgres-sions in southern Bolivia and Argentina. J. S. Am. Earth Sci.

[b28] Hoorn C (1993). Marine incursions and the influence of Andean tectonics on the Miocene depositional history of northwestern Amazonia – results of a palynostratigraphic study. Palaeogeogr. Palaeoclimatol. Palaeoecol.

[b29] Hoorn C, Wesselingh FP (2010). Amazonia: landscape and species evolution. A look into the past.

[b30] Howes GJ (1983). The cranial muscles of loricarioid catfishes, their homologies and value as taxonomic characters (Teleostei: Siluroidei). Bull. Br. Mus. Nat. Hist. Zool. Ser.

[b31] Hubert N, Renno JF (2006). Historical biogeography of South American freshwater fishes. J. Biogeogr.

[b32] Huelsenbeck JP, Ronquist F (2001). Mrbayes: bayesian inference of phylogenetic trees. Bioinformatics.

[b33] Isbrücker IJH (1980). Classification and catalogue of themailed Loricariidae (Pisces, Siluriformes). Versl. Tech. Gegevens. Instituut voor Taxonomische Zoölogie (Zoölogisch Museum) Universiteit van Amsterdam.

[b35] Langeani F (1990). Revisão do gênero *Neoplecostomus*, com a descrição de quatro espécies novas do sudeste brasileiro (Ostariophysi, Siluriformes, Loricariidae). Comunicações do Museu de Ciências da PUCRS, Série Zoologia.

[b36] Lovejoy NR, Bermingham E, Martin AP (1998). Marine incursion into South America. Nature.

[b37] Lundberg JG, Malabarba LR, Reis RE, Vari RP, Lucena CAS, Lucena ZMS (1998). The temporal context for diversification of Neotropical fishes. Phylogeny and classification of Neotropical fishes.

[b38] Lundberg JG (2005). *Brachyplatystoma promagdalena*, new species, a fossil goliath catfish (Siluriformes: Pimelodidae) from the Miocene of Colombia, South America. Neotrop. Ichthyol.

[b39] Lundberg JG, Aguilera O (2003). The late Miocene Phractocephalus catfish (Siluriformes: Pimelodidae) from Urumaco, Venezuela: additional specimens and reinterpretation as a distinct species. Neotrop. Ichthyol.

[b40] Lundberg JG, Chernoff B (1992). A Miocene fossil of the Amazonian fish Arapaima (Teleostei, Arapaimidae) from the Magdalena river region of Colombia – biogeographic and evolutionary implications. Biotropica.

[b41] Lundberg JG, Sullivan JP, Rodiles-Hernandez R, Hendrickson DA (2007). Discovery of African roots for the Mesoamerican Chiapas catfish, Lacantunia enigmatica, requires an ancient intercontinen-tal passage. Proc. Acad. Nat. Sci. Philadelphia.

[b42] Malabarba MCSL, Lundberg JG (2007). A fossil loricariid catfish (Siluriformes: Loricarioidea) from the Taubaté Basin, eastern Brazil. Neotrop. Ichthyol.

[b43] Malabarba LR, Malabarba MCSL (2008).

[b44] Miller KG, Kominz CAS, Browning JV, Wright DJ, Mountain GS, Katz ME (2005). The Phanerozoic record of global sea-level change. Science.

[b45] Monsch KA (1998). Miocene fish faunas from the northwestern Amazonia basin (Colombia, Peru, Brazil) with evidence of marine incursions. Palaeogeogr. Palaeoclimatol. Palaeoecol.

[b46] Montoya-Burgos JI (2003). Historical biogeography of the catfish genus *Hypostomus* (Siluriformes: Loricariidae), with implications on the diversification of Neotropical ichthyofauna. Mol. Ecol.

[b47] Montoya-Burgos JI, Muller S, Weber C, Pawlowski J, Malabarba LR, Reis RE, Vari RP, Lucena ZM, Lucena CAS (1998). Phylogenetic relationships of the Loricariidae (Siluriformes) basen on mitochondrial rRNA gene sequences. Phylogeny and classification of Neotropical fishes.

[b48] Müller D, Sdrolias M, Gaina C, Steinberger B, Heine C (2008). Long-term sea-level fluctuations driven by ocean basin dynamics. Science.

[b50] Pereira EHL (2005). Resurrection of *Pareiorhaphis* Miranda Ribeiro, 1918 (Teleostei: Siluriformes: Loricariidae), and description of a new species from the Rio Iguaçu Basin, Brazil. Neotrop. Ichthyol.

[b51] Pereira EHL, Reis RE (2002). Revision of the loricariid genera *Hemipsilichthys* and *Isbrueckerichthys* (Teleostei: Siluriformes) with descriptions of five new species of *Hemipsilichthys*. Ichthyol. Explor. Freshw.

[b52] Pereira EHL, Vieira F, Reis RE (2007). A new species of sexually dimorphic *Pareiorhaphis* Miranda Ribeiro, 1918 (Siluriformes: Loricariidae) from the Rio Doce Basin, Brazil. Neotrop. Ichthyol.

[b53] Pereira EHL, Vieira F, Reis RE (2010). *Pareiorhaphis scutula*, a new species of neoplecostomine catfish (Siluriformes: Loricariidae) from the upper rio Doce basin, Southeastern Brazil. Neotrop. Ichthyol.

[b54] Petit JR, Jouzel J, Raynaud D, Barkov NI, Barnola JM, Basile I (1999). Climate and atmospheric history of the past 420,000 years from the Vostok Ice Core.

[b55] Posada D, Crandall KA (1998). Modeltest: testing the model of DNA substitution. Bioinformatics.

[b56] Rahmstorf S (2003). Thermohaline circulation: the current climate. Nature.

[b57] Rambaut A, Drummond AJ (2007a). http://beast.bio.ed.ac.uk/Tracer.

[b58] Rambaut A, Drummond AJ (2007b). http://beast.bio.ed.ac.uk/TreeAnnotator.

[b59] Rebata HLA, Gingras MK, Räsänen ME, Barberi M (2006). Tidal-channel deposits on a delta plain from the Upper Miocene Nauta Formation, Maranon Foreland Sub-basin, Peru. Sedimentology.

[b60] Ree RH, Smith SA (2008). Maximum likelihood inference of geographic range evolution by dipersal, local extinction, and cladogenesis. Syst. Biol.

[b61] Ree RH, Moore BR, Webb CO, Donoghue MJ (2005). A likelihood framework for inferring the evolution of geographic range on phylogenetic trees. Evolution.

[b62] Regan CT (1904). A monograph of the fishes of the family Loricariidae. Trans. Zool. Soc. Lond.

[b63] Reis RE, Kullander SO, Ferraris CJ (2003). Check list of the freshwater fishes of South and Central America.

[b64] Reis RE, Pereira EHL, Armbruster JW (2006). Delturinae, a new loricariid catfish subfamily (Teleostei, Siluriformes), with revisions of Delturus and Hemipsilichthys. Zool. J. Linn. Soc.

[b65] Ribeiro AC (2006). Tectonic history and the biogeography of the freshwater fishes from the coastal drainages of eastern Brazil: an example of faunal evolution associated with a divergent continental margin. Neotrop. Ichthyol.

[b66] Ribeiro AC, Lima FCT, Riccomini C, Menezes NA (2006). Fishes of the Atlantic Rainforest of Boracéia: testimonies of the Quaternary fault reactivation within a Neoproterozoic tectonic province in Southeastern Brazil. Ichthyol. Explor. Freshw.

[b67] Riccomini C, Assumpção M (1999). Quaternary tectonics in Brazil. Episodes.

[b68] Roddaz M, Viers J, Brusset S, Baby P, Herail G (2005). Sediment provenances and drainage evolution of the Neogene Amazonian foreland basin. Earth Planet. Sci. Lett.

[b69] Ronquist F, Huelsenbeck JP (2003). MrBayes 3: bayesian phylogenetic inference under mixed models. Bioinformatics.

[b70] Rossetti DD (2001). Late Cenozoic sedimentary evolution in north-eastern Para, Brazil, within the context of sea level changes. J. S. Am. Earth Sci.

[b71] Rothman DH, Hayes JM, Summons RE (2003). Dynamics of the Neoproterozoic carbon cycle. Proc. Natl. Acad. Sci. USA 100.

[b72] Sanchez-Villagra MR, Aguilera OA (2006). Neogene vertebrates from Urumaco, Falcon State, Venezuela: diversity and signifi cance. J. Syst. Paleontol.

[b73] Santos RN, Ferreira EJG, Amadio S (2008). Effect of seasonality and trophic group on energy acquisition in Amazonian fish. Ecol. Freshw. Fish.

[b74] Schaefer SA (1987). Osteology of *Hypostomus plecostomus* (Linnaeus) with a phylogenetic analysis of the loricariid subfamilies (Pisces: Siluroidei). Nat. Hist. Mus. Los Angeles County.

[b75] Stamatakis A, Hoover P, Rougemont J (2008). A rapid bootstrap algorithm for the RAxML web-servers. Syst. Biol.

[b76] Suguio K, Martin L, Bittencourt ACS, Dominguez JML, Flexor JM, Azevedo AEG (1985). Flutuações do nível relativo do mar durante o Quaternário superior ao longo do litoral brasileiro e suas implicações na sedimentação costeira. Revista Brasileira de Geociências.

[b77] Swofford DL (2003). PAUP*: phylogenetic analysis using parsimony (*and other methods). Version 4.

[b78] Tamura K, Dudley J, Nei M, Kumar S (2007). Mega 4: molecular evolutionary genetics analysis (MEGA) software version 4.0. Mol. Biol. Evol.

[b80] Weitzman SH, Menezes NA, Weitzman MJ, Vanzolini PE, Heyer WR (1988). Phylogenetic biogeography of the glandulocaudini (Teleostei: Characiformes, Characidae) with comments on the distribution of other freshwater fishes in eastern and southeastern Brazil. Proceedings of a workshop on Neotropical distribution patterns.

[b81] Wilkinson MJ, Marshall LG, Lundberg JG (2006). River behaviour on megafans and potential influences on diversification and distribution of aquatic organisms. J. S. Am. Earth Sci.

[b82] Zachos JC, Dickens GR, Zeebe RE (2008). An early Cenozoic perspective on greenhouse warming and carbon-cycle dynamics. Nature.

[b83] Zawadzki CH, Pavanelli CS, Langeani F (2008). *Neoplecostomus* (Teleostei: Loricariidae) from the Upper Rio Paraná Basin, Brazil, with description of three new species. Zootaxa.

