# Supplementary material for: Evolutionary and biogeographic history of the subfamily Neoplecostominae (Siluriformes: Loricariidae)
Source: Ecol Evol. 2012 Aug 29;2(10):2438–49. doi: 10.1002/ece3.368 (PMC3492771; doi:10.1002/ece3.368)
Supplement: Supplementary file 1 [file ece30002-2438-SD1.docx]

**Supplementary Table 1.** Species included in the present study. LBP = Laboratório de Biologia e Genética de Peixes, Universidade Estadual Paulista. NUP = Núcleo de Pesquisas em Limnologia, Ictiologia e Aqüicultura, Universidade Estadual de Maringá. MHNG = Museum of Natural History of the City of Geneva. Asterisk indicates the geographic position of the city which the samples were collected and not the collection site.

| ***Number*** | ***Collection No.*** | **Fish No.** | **Genbank (16S/COI/CytB/12S/F-reticulon)** | **Species** | **Location (river, city, state, hydrographic Basin)** | ***Geographic Position*** |
| --- | --- | --- | --- | --- | --- | --- |
| **1** | **LBP2732** | 17441 | FJ434509/FJ434532/FJ965514/FJ965470/JN689302 | *Neoplecostomus paranensis* | Córrego Mocoquinha/Cajuru/SP/Rio Grande Basin | S 21°19'37'' W 47°14'19'' |
| **2** | **LBP2732** | 17444 | FJ965495/FJ965505/FJ965515/FJ965471/JN689303 | *Neoplecostomus paranensis* | Córrego Mocoquinha/Cajuru/SP/Rio Grande Basin | S 21°19'37'' W 47°14'19'' |
| **3** | **LBP6544** | 32377 | JN089838/JN089813/JN089789/JN089863/JN689305 | *Neoplecostomus* sp. 1 | Córrego Sem Nome/Ouro Fino/MG/Rio Grande Basin | S 22°11'36'' W 46°22'44'' |
| **4** | **LBP7469** | 33440 | JN089841/JN089816/JN089792/JN089866/JN689308 | *Neoplecostomus* sp. 2 | Córrego Espraiado/Borda da Mata/MG/Rio Grande Basin | S 22°16'27" W 46°09'47" |
| **5** | **LBP7462** | 33403 | JN089840/JN089815/JN089791/JN089865/JN689307 | *Neoplecostomus* sp. 3 | Córrego Monjolinho/S. Bto Sapucaí/MG/Rio Grande Basin | S 22°42'24'' W 45°42'57'' |
| **6** | **LBP6334** | 29832 | JN089839/JN089814/JN089790/JN089864/JN689306 | *Neoplecostomus* sp. 4 | Afluente do Rio Sapucaí/Campos do Jordão/SP/Rio Grande Basin | S 22°40'35'' W 45°33'34'' |
| **7** | **LBP2861** | 18616 | JN089833/JN089808/JN089784/JN089858/JN689298 | *Neoplecostomus bandeirante* | Rio Paraitinga/Salesópolis/SP/Rio Tietê Basin | S 23°31'37'' W 45°45'52'' |
| **8** | **LBP7467** | 33428 | JN089836/JN089811/JN089787/JN089861/JN689301 | *Neoplecostomus* sp. 5 | Casca D'antas/Rio Jaguarí/Rio Tietê/Rio Grande Basin | S 22°46'08'' W 45°59'25'' |
| **9** | **LBP4959** | 10248 | JN089832/JN089806/JN089783/JN089857/JN689296 | *Neoplecostomus* sp. 6 | Córrego do Sapateiro/Barbacena/MG/Rio Grande Basin | S 21°16'26'' W 43°38'37'' |
| **10** | **LBP7466** | 33421 | JN089835/JN089810/JN089786/JN089860/JN689300 | *Neoplecostomus* sp. 7 | Córrego Tamborete/Furnas/MG/Rio Grande Basin | S 20°38'54'' W 46°09'52'' |
| **11** | **LBP5901** | 27990 | JN089837/JN089812/JN089788/JN089862/JN689304 | *Neoplecostomus langeanii* | Córrego São Domingos/Muzambinho/MG/Rio Grande Basin | S 21°17'37'' W 46°29'06'' |
| **12** | **LBP6426** | 29991 | JN089834/JN089809/JN089785/JN089859/JN689299 | *Neoplecostomus* sp. 8 | Rio Mourão/Campo Mourão/PR/Rio Tibagi Basin | S 24°06'16'' W 52°19'31'' |
| **13** | **LBP1096** | 10268 | JN089831/JN089806/JN089782/JN089856/JN689294 | *Neoplecostomus* sp. 9 | Rio Chopotó/Desterro de Melo/MG/Rio Doce Basin | S 21°08'56'' W 43°23'58'' |
| **14** | **LBP709** | 6049 | JN089842/JN089817/JN089793/JN089867/JN689309 | *Neoplecostomus* cf. *botucatu* | Córrego Hortelã/Botucatu/SP/Rio Paranapanema Basin | S 22°55'00'' W 48°30'00'' |
| **15** | **LBP7525** | 34832 | JN089843/JN089818/JN089794/JN089868/- | *Neoplecostomus botucatu* | Cachoeira Véu da Noiva/Botucatu/SP/Rio Paranapanema Basin | S 22°59'25" W 48°25'37" |
| **16** | **NUP3560** | 9701 | FJ434506/FJ434529/FJ965516/FJ965472/JN689293 | *Neoplecostomus yapo* | Rio Tibagi/Fortaleza/PR/Rio Paranapanema Basin | S 24°25'30'' W 50°13'55'' |
| **17** | **LBP645** | 7593 | FJ434508/FJ434531/FJ965518/FJ965484/JN689311 | *Neoplecostomus microps* | Ribeirão Cajarana/Pindamonhangaba/SP/Rio Paraíba do Sul Basin | S 22°46'00'' W 45°27'00'' |
| **18** | **LBP894** | 9735 | JN089844/JN089819/JN089795/JN089869/JN689322 | *Neoplecostomus ribeirensis* | Rio Iporanga/Iporanga/SP/Rio Ribeira do Iguape Basin | S 23°26'23" W 47°25'26"* |
| **19** | **LBP7384** | 34837 | JN089845/JN089820/JN089796/JN089870/JN689323 | *Neoplecostomus ribeirensis* | Rio Água Doce/Tapiraí/SP/Rio Ribeira do Iguape Basin | S 22°27'02'' W 49°14'26'' |
| **20** | **LBP2551** | 15243 | FJ434507/FJ434530/ FJ965512/FJ965485/JN689312 | *Neoplecostomus espiritosantensis* | Rio Jucu/Domingos Martins/ES/Oriental Coastal Basin | S 22°49'51'' W 44°51'53'' |
| **21** | **NUP2528** | 9423 | FJ434520/FJ434543/FJ965513/FJ965469/JN689295 | *Neoplecostomus corumba* | Rio Corumbá/GO/Rio Paranaíba Basin/Upper Rio Paraná Basin | S 17°43'37'' W 48°32'54'' |
| **22** | **LBP6537** | 31681 | FJ965494/FJ965504/FJ965519/FJ965483/JN689313 | *Neoplecostomus franciscoensis* | Rio das Velhas/Brumadinho/MG/Rio São Francisco Basin | S 20°00'37'' W 43°58'08'' |
| **23** | **LBP7383** | 34843 | FJ965496/FJ965507/FJ965517/FJ965473/JN689310 | *Neoplecostomus selenae* | Ribeirão das Batéias/Riacho Grande/SP/Rio Paranapanema Basin | S 24°12'02'' W 48°25'06'' |
| **24** | **LBP7472** | 32387 | JN089822/JN089798/JN089773/JN089847/JN689284 | New genus and species 1 | Córrego Guarda Mor/Guarda Mor/MG/Rio São Francisco Basin | S 17°46'18'' W 47°05'43'' |
| **25** | **LBP902** | 7989 | FJ434514/FJ434537/FJ965532/FJ965480/JN689319 | *Pareiorhaphis steindachneri* | Rio Itapucu/Jaraguá do Sul/SC/Oriental Coastal Basin | S 26°26'48'' W 49°09'54'' |
| **26** | **LBP1161** | 8935 | FJ434512/FJ434535/FJ965530/FJ965479/JN689320 | *Pareiorhaphis vestigipinnis* | Rio Caveiras/Painel/SC/Rio Uruguai Basin | S 27°55'12'' W 50°06'25'' |
| **27** | **LBP701** | 7363 | FJ434513/FJ434536/FJ965531/FJ965482/JN689321 | *Pareiorhaphis hystrix* | Rio Tainhas/Tainhas/RS/Oriental Coastal Basin | S 29°24'06'' W 50°27'01'' |
| **28** | **LBP748** | 8257 | FJ625811/FJ625820/FJ965524/FJ965481/JN689318 | *Pareiorhaphis splendens* | Rio São João/Guaruva/PR/Oriental Coastal Basin | S 25°58'39'' W 48°52'59'' |
| **29** | **LBP7373** | 34853 | FJ965490/FJ965506/FJ965525/FJ965477/JN689297 | *Isbrueckerichthys alipionis* | Rio Betari/Iporanga/SP/Rio Ribeira do Iguape Basin | S 24°33'42'' W 48°40'05'' |
| **30** | **LBP6427** | 29996 | FJ965488/FJ965497/FJ965527/FJ965476/JN689281 | *Isbrueckerichthys* sp. 1 | Rio Charqueada/Campo Mourão/PR/Rio Paranapanema Basin | S 24°02'45" W 52°22'59"* |
| **31** | **LBP7385** | 34852 | FJ965491/FJ965502/FJ965526/FJ965478/JN689324 | *Isbrueckerichthys epakmos* | Rio Água Doce/Tapiraí/SP/Rio Ribeira do Iguape Basin | S 22°27'02'' W 49°14'26'' |
| **32** | **LBP6389** | 29765 | FJ965489/FJ965503/FJ965529/FJ965474/JN689326 | *Isbrueckerichthys* cf. *calvus* | Rio Taquará/California/PR/Rio Paranapanema Basin | S 23°40'55'' W 51°18'55'' |
| **33** | **LBP2650** | 17402 | FJ625812/FJ625821/FJ965528/FJ965475/JN689325 | *Isbrueckerichthys duseni* | Rio Pulador/Campinhos/PR/Rio Ribeira de Iguape Basin | S 25°02'47'' W 49°05'34'' |
| **34** | **LBP1766** | 12886 | FJ965493/FJ965501/FJ965521/FJ965466/JN689317 | *Kronichthys* sp. 1 | Rio Sítio do Meio/Mongaguá/SP/Oriental Coastal Basin | S 24°05'11'' W 46°43'59'' |
| **35** | **LBP795** | 8304 | FJ434503/FJ434526/FJ965522/FJ965463/JN689315 | *Kronichthys lacerta* | Rio Marumbi/Morretes/PR/Oriental Coastal Basin | S 25°29'12'' W 48°49'58'' |
| **36** | **LBP2122** | 15096 | FJ434502/FJ434525/FJ965520/FJ965464/JN689314 | *Kronichthys heylandi* | Rio Parati-Mirim/Parati/RJ/Oriental Coastal Basin | S 25°29'12'' W 48°49'58'' |
| **37** | **LBP515** | 6334 | FJ965492/FJ965500/FJ965523/FJ965465/JN689316 | *Kronichthys subteres* | Rio Betari/Iporanga/SP/Rio Ribeira do Iguape Basin | S 24°33'44'' W 48°40'10'' |
| **38** | **LBP4391** | 24189 | FJ434517/FJ434540/FJ965510/FJ965462/JN689280 | *Pareiorhina* cf. *rudolphi* | Ribeirão Guaxinduva/Jundiaí/SP/Rio Tietê Basin | S 23°15'00'' W 46°58'00'' |
| **39** | **LBP8368** | 37559 | JN089829/JN089804/JN089780/JN089854/JN689291 | *Pareiorhina* *carrancas* | Córrego Beijinho/Carrancas/MG/Rio Grande Basin | S 21°26'39'' W 44°36'08'' |
| **40** | **LBP8368** | 37560 | JN089830/JN089805/JN089781/JN089855/JN689292 | *Pareiorhina carrancas* | Córrego Beijinho/Carrancas/MG/Rio Grande Basin | S 21°26'39'' W 44°36'08'' |
| **41** | **LBP1087** | 10256 | JN089828/JN089803/JN089779/JN089853/JN689290 | *Pareiorhina* cf. *carrancas* | Córrego do Sapateiro/Barbacena/MG/Rio Grande Basin | S 21°16'26'' W 43°38'36'' |
| **42** | **LBP8380** | 37564 | JN089826/JN689277/JN089777/JN089851/JN689288 | *Pareiorhina* sp. 1 | Rio Pomba/Santa Barbara do Tugúrio/MG/Rio Paraíba do Sul Basin | S 21°14'07'' W 43°30'50'' |
| **43** | **LBP8380** | 37565 | JN089827/JN089802/JN089778/JN089852/JN689289 | *Pareiorhina* sp. 1 | Rio Pomba/Santa Barbara do Tugúrio/MG/Rio Paraíba do Sul Basin | S 21°14'07'' W 43°30'50'' |
| **44** | **LBP8044** | 37775 | JN089824/JN089800/JN089775/JN089849/- | *Pareiorhina rudolphi* | Ribeirão Piquete/Piquete/SP/Rio Paraíba do Sul Basin | S 22°35'00'' W 45°10'08'' |
| **45** | **LBP8347** | 37571 | JN089825/JN089801/JN089776/JN089850/JN689287 | New genus and species 2 | Rio Piçarrão/Ferros/MG/Rio Doce Basin | S 19º40'53'' W 43º00'50'' |
| **46** | **LBP616** | 7564 | FJ625810/FJ625819/FJ965511/FJ965461/JN689286 | *Pseudotocinclus juquiae* | Rio Juquiá/Juquitiba/SP/Rio Ribeira do Iguape Basin | S 23°59'49'' W 46°56'01'' |
| **47** | **LBP2931** | 18994 | JN089823/JN089799/JN089774/JN089848/JN689285 | *Pseudotocinclus tietensis* | Rio Paraitinga/Salesópolis/SP/Rio Tietê Basin | S 23°31'36'' W 45°49'11'' |
| **48** | **LBP2001** | 12191 | FJ965487/FJ965499/FJ965509/FJ965499/- | *Corumbataia cuestae* | Rio Alambari/Botucatu/SP/Rio Tietê Basin | S 22°56'08'' W 48°19'15'' |
| **49** | **MHNG2678.015** | PR12 | FJ965486/FJ965498/FJ965508/FJ965468/JN689282 | *Hypoptopoma inexpectatum* | Rio Paraná/Santa Fé/Argentina | Not available |
| **50** | **LBP3510** | 21309 | FJ625809/FJ625818/FJ965533/FJ965458/JN689283 | *Hypostomus nigromaculatus* | Córrego Hortelã/Botucatu/SP/Rio Paranapanema Basin | S 22°56'28'' W 48°35'03'' |
| **51** | **LBP730** | 8369 | JN089846/JN089821/JN089797/JN089871/- | *Rineloricaria jaraguensis* | Córrego Ribeirão Cavalo/Jaraguá do Sul/SC/- | S 26°28'15'' W 49°10'57'' |
| **52** | **LBP2368** | 15363 | FJ434499/FJ434524/FJ965535/FJ965460/JN689278 | *Hemipsilichthys gobio* | Rio Macaquinho/Bairro dos Macacos/SP/Rio Paraíba do Sul Basin | S 22°49'51'' W 44°51'53'' |
| **53** | **LBP4956** | 10241 | FJ625808/FJ625817/FJ965534/FJ965459/JN689279 | *Hemipsilichthys papillatus* | Ribeirão da Jacutinga/Bom Jardim de Minas/MG/Rio Paraíba do Sul Basin | S 22°02'27'' W 44°09'43'' |
| **54** | **NM-2010** | 909838 | AP012004 (GenBank complete mitochondrial genome) | *Astroblepus* sp. | Not available | Not available |

**Supplementary Table 2.** Primers used in the present study to amplify partial sequences of 12S and 16S rRNA, cytochrome oxidase subunit I (COI), cytochrome B (CytB) and F-reticulon 4.

| **Region and Fragment Length** | **Name** | **References** | **Primer Sequence** |
| --- | --- | --- | --- |
| **F-reticulon 4 (1900 bp)** | Freticul4-D | Chiachio *et al.* (2008) | 5’-AGG CTA ACT CGC TYT SGG CTT TG-3’ |
|  | Freticul4-R |  | 5’-GGC AVA GRG CRA ART CCA TCT C-3’ |
|  | Freticul4 D2 |  | 5’-CTT TGG TTC GGA ATG GAA AC-3’ |
|  | Freticul4 R2 |  | 5’-AAR TCC ATC TCA CGC AGG A-3’ |
|  | Freticul4 iR |  | 5’-AGG CTC TGC AGT TTC TCT AG-3’ |
| **12S rRNA (900 bp)** | Phe-L941 | Present study | 5’-AAA TCA AAG CAT AAC ACT GAA GAT G-3’ |
|  | Val-H2010 |  | 5’-CCA ATT TGC ATG GAT GTC TTC TCG G-3’ |
| **16S rRNA (700 bp)** | 16Sar | Kocher *et al.* (1989) | 5’-ACG CCT GTT TAT CAA AAA CAT-3’ |
|  | 16Sbr |  | 5’-CCG GTC TGA ACT CAG ATC ACG T-3’ |
| **COI (700 bp)** | FishF1 | Ward *et al.* (2005) | 5’-TCA ACC AAC CAC AAA GAC ATT GGC AC-3’ |
|  | FishR1 |  | 5’-TAG ACT TCT GGG TGG CCA AAG AAT CA-3’ |
| **CytB (900 bp)** | L14841 | Oliveira *et al.* (2011) | 5’-CCA TCC AAC ATC TCA GCA TGA TGA AA 3’ |
|  | H15915b |  | 5’-AAC CTC CGA TCT TCG GAT TAC AAG AC 3’ |

**Supplementary Table 3.** Nucleotide substitution models for each partition used in the phylogenetic analyses of each program.

| **Gene** | **Modeltest**  **Search** | **Maximum Likelihood**  **RAxML Analysis** | **Bayesian Inference with**  **Mrbayes v.3.0** | **Molecular Clock Analysis**  **with Beast v.1.6.2** | **Bases** |
| --- | --- | --- | --- | --- | --- |
| **12S** | HKY+I+G | GTR+G | lset nst 2+I+G | HKY+I+G | 1 – 754 |
| **16S** | HKY+G | GTR+G | lset nst 2+G | HKY+G | 755 – 1275 |
| **CytB** |  |  |  |  | 1276 – 2067 |
| **CytB first base of codon** | K80+G | GTR+G | lset nst 2+G | HKY+G | 1276 – 2067 \3 |
| **CytB second base of codon** | HKY+G | GTR+G | lset nst 2+G | HKY+G | 1277 – 2067 \3 |
| **CytB third base of codon** | TN+G | GTR+G | lset nst 1+G | TN93+G | 1278 – 2067 \3 |
| **COI** |  |  |  |  | 2068 – 2601 |
| **COI first base of codon** | TNef+G | GTR+G | lset nst 1+G | TN93+G | 2068 – 2601 \3 |
| **COI second base of codon** | F81 | GTR+G | lset nst 1 | HKY | 2069 – 2601 \3 |
| **COI third base of codon** | TN+G | GTR+G | lset nst 1+G | TN93+G | 2070 – 2601 \3 |
| **F-reticulon** |  |  |  |  |  |
| **F-reticulon exon 1** |  |  |  |  | 2602 – 2688 |
| **F-retex1 first base of codon** | JC | GTR+G | lset nst 1 | HKY | 2602 – 2688 \3 |
| **F-retex1 second base of codon** | F81 | GTR+G | lset nst 1 | HKY | 2603 – 2688 \3 |
| **F-retex1 third base of codon** | JC | GTR+G | lset nst 1 | HKY | 2604 – 2688 \3 |
| **F-reticulon intron 1** | HKY+G | GTR+G | lset nst 2+G | HKY+G | 2689 – 3585 |
| **F-reticulon exon 2** |  |  |  |  | 3586 – 3786 |
| **F-retex2 first base of codon** | JC | GTR+G | lset nst 1 | HKY | 3586 – 3786 \3 |
| **F-retex2 second base of codon** | K80+G | GTR+G | lset nst 2+G | HKY+G | 3587 – 3786 \3 |
| **F-retex2 third base of codon** | K80 | GTR+G | lset nst 2 | HKY | 3588 – 3786 \3 |
| **F-reticulon intron 2** | HKY+G |  | lset nst 2+G | HKY+G | 3787 – 4065 |
| **F-reticulon exon 3** |  |  |  |  | 4066 – 4676 |
| **F-retex3 first base of codon** | F81+G | GTR+G | lset nst 1+G | HKY+G | 4066 – 4676 \3 |
| **F-retex3 second base of codon** | HKY+G | GTR+G | lset nst 2+G | HKY+G | 4067 – 4676 \3 |
| **F-retex3 third base of codon** | F81+G | GTR+G | lset nst 1+G | HKY+G | 4068 – 4676 \3 |
